# Shorter Telomere Length in Individuals with Neurocognitive Disorder and *APOE* ε4 Genotype

**DOI:** 10.3390/ijms26104577

**Published:** 2025-05-10

**Authors:** Paola Mejía-Ortiz, Alma Delia Genis-Mendoza, Ramon Ramírez Villanueva, Susana López Ramírez, Rafael Guzmán Sánchez, Thalia Fernández, Jorge Sigg-Alonso, Humberto Nicolini-Sánchez

**Affiliations:** 1 Posgrado en Ciencias (Neurobiología), Unidad de Enseñanza Instituto de Neurobiología, Universidad Nacional Autónoma de México, Campus Juriquilla 3001, Querétaro C.P. 76230, Mexico; paome.oz@gmail.com; 2 Laboratorio de Genómica de las Enfermedades Psiquiátricas y Neurodegenerativas, Instituto Nacional de Medicina Genómica, Secretaría de Salud, Ciudad de México C.P. 14610, Mexico; adgenis@inmegen.gob.mx; 3 Hospital Psiquiátrico Infantil Dr. Juan N. Navarro, Servicios de Atención Psiquiátrica, Secretaria de Salud, Periferico sur 4809, Tlalpan CDMX, Ciudad de México C.P. 14610, Mexico; 4 Servicio de Geriatría y Gerontología, ISSSTE Clínica de Medicina Familiar Dr. Ignacio Chávez, Oriental 10, Coapa, Coyoacán, Ciudad de México C.P. 04800, Mexico; ramirezramon1995@gmail.com (R.R.V.); shanyx_87@hotmail.com (S.L.R.); medicorafa@gmail.com (R.G.S.); 5 Laboratorio de Psicofisiología, Departamento de Neurobiología Conductual y Cognitiva, Instituto de Neurobiología, Universidad Nacional Autónoma de México, Campus Juriquilla 3001, Querétaro C.P. 76230, Mexico; thaliafh@yahoo.com.mx (T.F.); jorgesigg@gmail.com (J.S.-A.)

**Keywords:** neurocognitive disorder, telomere length, *APOE*, cognitive impairment, cognitive reserve

## Abstract

Neurocognitive disorders (NCD) are neurodegenerative diseases characterized by decline or loss of cognitive functions. Aging and the *APOE* genotype have been identified as major risk factors. Telomere length (TL) has been proposed as a biomarker of aging, with shorter TL associated with cognitive decline. This study investigated the relationship between TL and the *APOE* genotype in individuals with cognitive impairments (CIs). A total of 170 participants aged >55 years were included. Cognitive function was assessed using the MMSE and MoCA tests. Relative telomere quantification and *APOE* genotype were determined by real-time PCR. A significant association was observed between shorter TL and an increased risk of CI (*p* < 0.001). Although *APOE* ε4 is a known genetic risk factor, its association with CI was less clear in this study population, as a considerable proportion of ε4 carriers did not present cognitive impairment (*p* < 0.05). However, ε4 carriers with CI tended to have shorter TL than those with non-cognitive impairment (NCI-SMC). Furthermore, fewer years of education were strongly correlated with higher CI risk (*p* < 0.0001). Overall, individuals with both shorter telomeres and lower educational levels exhibited the highest risk of CI. *APOE* ε4 may contribute to telomere shortening.

## 1. Introduction

Neurocognitive disorders (NCD) are multifactorial diseases characterized by a loss of cognitive abilities beyond those expected in normal aging [1,2,3]. The main etiology identified is Alzheimer’s disease (AD), with 80% of all cases and an incidence of 1.5 million in the world. Vascular dementia is the second most common cause of NCD. Regardless of etiology, the main risk factors were age and *APOE* genotype [4,5,6].

The *APOE* genotype is one of the most important markers of risk for Alzheimer’s disease [3]; there are three isoforms that represent a higher or lower risk in the development of Alzheimer’s disease: *ε2* (protecting allele), *ε3* (neutral), and *ε4* (highest risk) [7]. Sufficient evidence establishes that the presence of the *ε4* allele in this gene confers a high risk of developing NCD [8]. These findings are related to animal models, clinical studies, and GWAS analyses [9,10].

Originally, the *APOE* gene was identified by linkage studies accounting for 15–20% of inheritance. Namba and coworkers in 1991 first reported *APOE* reactivity in amyloid plaques and established its link to brain and amyloidogenic diseases [11]. A few years later, Allen Roses et al. (1994) established a connection to Alzheimer’s disease [12]. Findings regarding the *APOE*-dependent interaction with AB deposition in amyloid plaques have been consistently replicated in both animal and cellular models [13].

Likewise, in vitro experiments on AB peptide binding to membranes and its association with the *APOE* ε4 genotype were performed, finding that the ε4 allele is a strong genetic risk factor for AD and amyloid deposition [7].

Immunohistochemistry assays were developed to detect β-amyloid protein deposition in the brain tissues associated with different *APOE* alleles. These findings established a link between β-amyloid accumulation, *APOE* genotype, and Alzheimer’s disease, which was later corroborated by larger population studies [14,15]. Recent studies have replicated these findings using different methodologies; a significant interaction between Aβ 42/40 amyloid load and *APOE* ε4 has been supported by PET studies [16], evidencing a differential accumulation between different genotypes. The mechanisms linking this pathological process with Alzheimer’s disease have suggested that a loss of brain volume and cortical atrophy impair cognitive functions, as well as higher levels of inflammatory markers [16,17,18].

Currently, three large genome-wide association studies (GWAS) have identified the *APOE* locus [10,19]. In 2013, the International Alzheimer’s Genomics Project conducted a meta-analysis of all GWAS, the largest conducted for the disease until 2018, which included data from four consortia: Alzheimer’s Disease Genetics Consortium (ADGC), Cohort Consortium for Heart and Aging Research in Genomic Epidemiology (CHARGE), the European Alzheimer’s Disease Initiative (EADI), and the Genetic and Environmental Risk in Alzheimer’s Disease (GERAD) Consortium [10]. In 2019, Kunkle et al. analyzed data from 94,437 individuals, cases, and controls using the IGAP consortium, confirming 20 risk loci in addition to *APOE* [20].

The latest and largest GWAS performed so far was conducted by Bellenguez et al. In their meta-analysis, they used data from the European Alzheimer’s and Dementia Biobank Consortium, which collected data from 20,464 clinically diagnosed AD cases and 22,244 controls from 15 European countries. In addition to *APOE*, 75 other risk loci were also identified. All three studies performed pathway enrichment analyses to identify pathways related to lipid, cholesterol, and lipoprotein processing; amyloid pathways; tau; and immunity, which connect to the role of the *APOE* gene in lipid metabolism and Alzheimer’s disease [21].

Research involving the *APOE* genotype is still ongoing, as it is important to increase the analyses in different types of populations, as most of them have been performed in European or Asian populations, in addition to the search for new associations that contribute to *APOE*-mediated pathogenesis. As supported by these studies, *APOE* has emerged as the most crucial risk factor, particularly in European populations [9,19].

Recently, the study of NCD has taken a multifactorial direction, including research involving an increasing number of regulatory mechanisms inherent to aging [16,18], such as metabolic, cellular, and genetic modifications [22,23,24]. Biological aging is a process of biological changes at the molecular, genomic, and cellular levels due to the accumulation of damage over time, leading to loss of physiological functions and increased catabolism [25]. The changes that occur during this process alter homeostasis, where self-regulation and regeneration are diminished, thereby affecting the proper functioning of organs and tissues [26,27].

Multiple mechanisms are associated with aging, including genomic instability, telomere attrition, epigenetic alterations, loss of proteostasis, impaired macroautophagy, dysregulated nutrient sensing, mitochondrial dysfunction, cellular senescence, stem cell depletion, altered intercellular communication, chronic inflammation, and dysbiosis [24,28].

In recent studies on neurodegenerative diseases, telomeric shortening has been identified as a biomarker of biological aging [29]. Telomeres, which consist of tandemly repeated sequences of non-coding DNA, are linked to proteins that protect the chromosome ends. These sequences are crucial for maintaining stability and determining the lifespan of cells and act as markers of both biological age and the aging process [29,30]. During mitotic divisions, incomplete replication of DNA in the telomeric region gradually shortens its length, resulting in replicative senescence [28,30]. This is a terminal state where cells stop proliferating, even when the necessary stimuli and growth conditions are present [30,31].

Decreased telomere length (TL) in humans has been associated with vascular and neurodegenerative diseases in the elderly population [29,32]. Some researchers have proposed that telomere length may be linked to hippocampal volume and cognitive function, including memory and executive abilities [33,34]. Despite progress in the study of the association between telomere length and cognitive status, some studies have found no significant evidence, such as the case of Demanelis et al., who found that telomere length is related to events present in aging but not to cognitive impairment [35,36].

Aging is a process characterized by interindividual differences, not only at the structural, metabolic, and chemical levels that influence brain changes but also in the ability to compensate for loss of function associated with brain damage, lifestyle, and genetic determinants [6,37,38].

## 2. Results and Discussion

The mean age observed was 70.5 ± 9.22 years; 44.7% (*n* = 76) were men, and 55.3% (*n* = 94) were women. The average number of years of education was 13.14 ± 7.16. Among all individuals, 58.2% (*n* = 99) had cognitive impairment (CI), whereas 41.8% (*n* = 71) had non-cognitive impairment with subjective memory complaints (NCI-SMC). No statistically significant differences were found between males and females in terms of years of education (mean ± SD: 13.22 ± 6.00 for males, 13.06 ± 7.99 for females) or age (71.61 ± 8.34 for males, 69.59 ± 9.83 for females) between the two groups. However, for the relationship between cognitive impairment and sex, statistically significant differences were found using the chi-square test (χ^2^ = 6.758, *p* = 0.009), and a higher proportion of males with cognitive impairment was observed than females (Table 1).

Several studies have reported that the prevalence of AD is higher in women than in men [39]. However, findings vary, as some authors indicate that this increased incidence is primarily observed in individuals aged 80–85 years or older, with a more pronounced increase in women as age advances.

These findings are complex, as some studies suggest that mild cognitive impairment (MCI) is more prevalent in men than in women, whereas advanced-stage dementia is more common in women. Mutchie et al. (2022) analyzed cognitive status in older adults with hip fractures and determined that men were more likely to have cognitive impairment when assessed using neuropsychological tests rather than clinical evaluations [40].

It is essential to consider the factors that influence cognitive impairment, such as comorbidities (e.g., hypertension, diabetes, hypercholesterolemia, obesity, and cardiovascular disease) [41]. Psychiatric conditions also play an important role. A study analyzing the impact of anxiety and depression on cognitive impairment found that men with anxiety had higher levels of cognitive impairment, whereas women with anxiety exhibited higher functional performance in daily activities. Conversely, men with depression showed lower cognitive impairment and better performance in daily activities [42].

### 2.1. Education and Cognitive Impairment

As shown in Table 1, there were differences in educational levels between the two groups (CI and NCI-SMC). Logistic regression analysis indicated that each additional year of education was associated with a 15.36% decrease in odds of cognitive impairment. (OR = 0.77, CI = 0.70 and 0.83, *p* < 0.0001).

However, age differences were also observed between the groups (W = 1521, *p* < 0.001), with individuals with CI being significantly older on average than those with NCI-SMC, suggesting that there may be an interaction between age and years of study that modifies the association with cognitive impairment.

**Figure 1:**
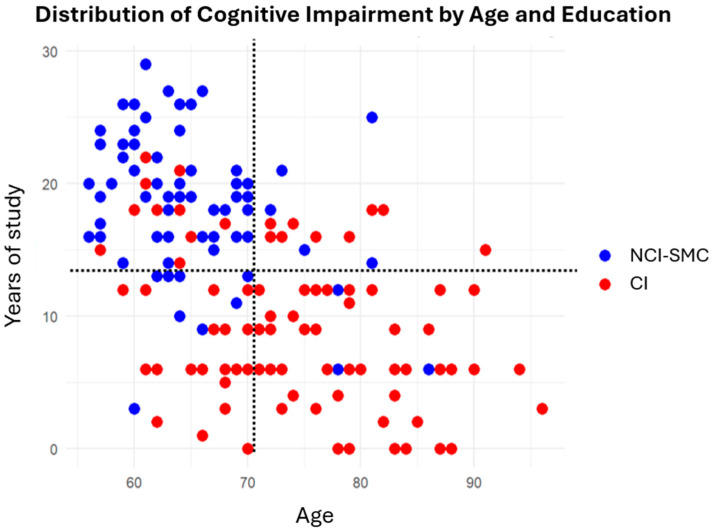
Distribution of cognitive impairment by age and education: cluster patterns. The dashed vertical line represents the mean age of the sample (mean = 70.5), and the dashed horizontal line indicates the mean number of years of education (mean = 12.56). Red dots represent individual CIs, while blue dots represent NCI-SMCs.

Therefore, an exploratory cluster analysis was performed to evaluate potential patterns in the joint distribution of age, years of education, and cognitive decline (Figure 1). The analysis showed possible age-related heterogeneity, with most individuals with NCI-SMC being younger and having more years of education. Since the cognitive reserve hypothesis proposes that a higher level of education may delay the onset of clinical signs, this finding suggests that the participants in this study, by virtue of their age and the cumulative effect of years of education, have not yet presented symptoms. Alternatively, the apparent protective effect of education may be confounded by age [43].

This hypothesis suggests that greater cognitive reserve, including higher educational attainment, contributes to more effective functional compensation in response to brain changes, resulting in a prolonged cognitive trajectory prior to the clinical onset of symptoms without necessarily altering the overall likelihood of developing impairment [43,44]. Some studies on cognitive reserve in Alzheimer’s patients have shown that higher cognitive reserve scores correlate with better cognitive performance; however, it did not prevent disease progression; on the contrary, some studies suggest that individuals with greater cognitive reserve may experience a faster rate of cognitive decline once symptoms emerge [45,46].

However, this remains a controversial issue, as some studies have found no conclusive evidence to support either side of this argument. For instance, a UK cohort study by Wilson et al. found that although a higher educational level was associated with better initial cognitive performance, it was not related to a later onset of cognitive decline, slower progression of dementia, or reduced incidence of neurodegenerative diseases. Therefore, the authors concluded that educational attainment primarily influences premorbid cognitive functioning without affecting the trajectory of cognitive aging or the course of the disease [47].

On the other hand, other studies have reported findings consistent with the protective role of cognitive reserve. For example, a UK cohort study by Yan found that a high cognitive reserve was associated with a slower decline in global cognitive function, memory, and reaction time. Similarly, studies conducted in Chinese populations have shown that higher baseline global cognition is associated with a slower annual rate of cognitive decline [48,49].

Based on cluster observations, a logistic regression model was used to analyze the interaction between years of education and age, with the aim of exploring whether the protective effect of education is stronger in younger individuals and diminishes with age. However, statistical results indicated that the effect of education did not vary with age in this dataset (OR = 0.176; 95% CI: 0.000002–13,592.3; *p* = 0.763).

**Figure 2:**
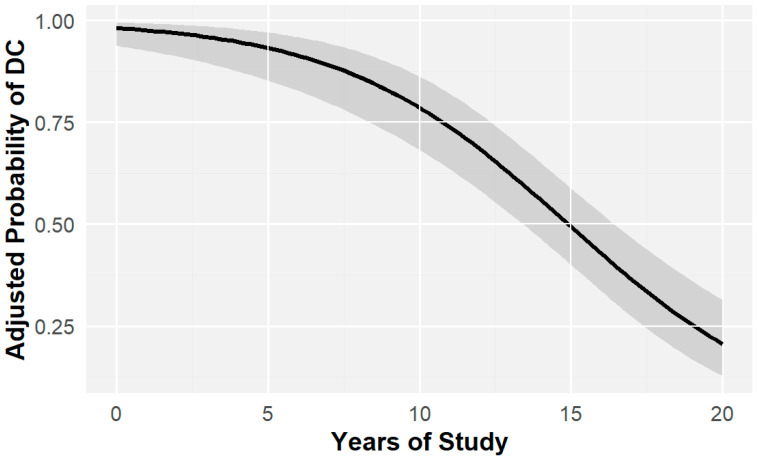
Probability of cognitive impairment by years of study adjusted for age. Risk of cognitive impairment as a function of age-adjusted number of years of study. The shaded area indicates the CI of the function. OR = 0.768, CI = 0.71–0.82, *p* < 0.0001.

Thus, years of education as a proxy for cognitive reserve can be considered an independent factor that influences the likelihood of cognitive decline. However, because age also affects cognitive deterioration, as detailed below, the logistic regression model was adjusted for age to control for and minimize potential confounders. This adjustment enabled a more precise estimation of the independent effect of educational level on cognitive decline. The association remained statistically significant with no substantial changes in the coefficients (OR = 0.768, IC = 0.71 and 0.82, *p* < 0.0001), indicating an inverse relationship between educational attainment and the likelihood of decline (Figure 2).

The concept of cognitive and brain reserves suggests that differences in individuals’ susceptibility to age-related brain changes or pathological processes are influenced by life experiences [43,48]. Studies have indicated that the incidence and prevalence of dementia decrease in individuals with larger brain volumes, more neurons, and greater synaptic density [43].

Animal studies have supported this concept by showing that enriched environments promote neuronal growth and new synaptic connections, thereby enhancing brain plasticity. Years of education, occupation, and cognitive engagement are commonly used as indicators of cognitive reserve, and higher education levels are associated with greater cognitive resilience [43].

Cognitive reserve has been proposed to be associated with a lower risk of cognitive impairment; however, research has shown contradictory results, largely because of the complex influence of occupation, life experiences, and social participation on cognitive development and stimulation [42,50]. While educational attainment reflects cognitive activity primarily during early life, individuals spend approximately 40–50% of their lives engaged in work, where varying levels of occupational complexity contribute to individual differences in cognitive development across the lifespan. Some studies have suggested that the development of activities and/or paid work with greater complexity in mental abilities has an advantage for cognitive health [51].

Both occupational activities and recreational or social participation activities are categorized according to their complexity to evaluate their relationship with cognitive performance in adults, focusing on comparing the degree of occupational complexity with cognitive states [52]. This shows that the development of activities with greater cognitive demand has advantages in global cognitive health in domains such as executive function and verbal episodic memory [53].

In addition, the complexity of the occupation is not the only factor present in the life of working individuals, since a job can demand high mental development and, in turn, generate environments of psychological stress that trigger pathological behaviors such as anxiety or depression. Similarly, factors such as exposure to occupational hazards or high physical demands can affect functional domains and reduce the ability to perform basic and advanced activities of daily living, which are associated with worse cognitive states in neurodegenerative diseases. Thus, it can be appreciated that cognitive reserve is a complex concept that encompasses numerous factors and life experiences [54].

Although this was a cross-sectional study and only years of education were considered, the observed risk suggests that at least one of the activities that contributes to cognitive reserve influences the incidence of cognitive decline, regardless of age. It is important to recognize that educational attainment reflects the development of mental abilities during critical stages of neurobiological development [55]. However, formal education typically ends decades before the onset of old age, whereas cognitive activity later in life may shape this trajectory and is associated with the rate of cognitive change. As previously noted, other life experiences in adulthood and old age, such as social engagement, cognitively demanding occupations, and interpersonal interactions, also play a significant role in cognitive aging [56]. Therefore, it is essential to complement these findings with cognitive reserve data that include a broader range of indicators to more accurately attribute the benefits of cognitive stimulation across the lifespan [55,56].

### 2.2. Age and Cognitive Impairment

**Figure 3:**
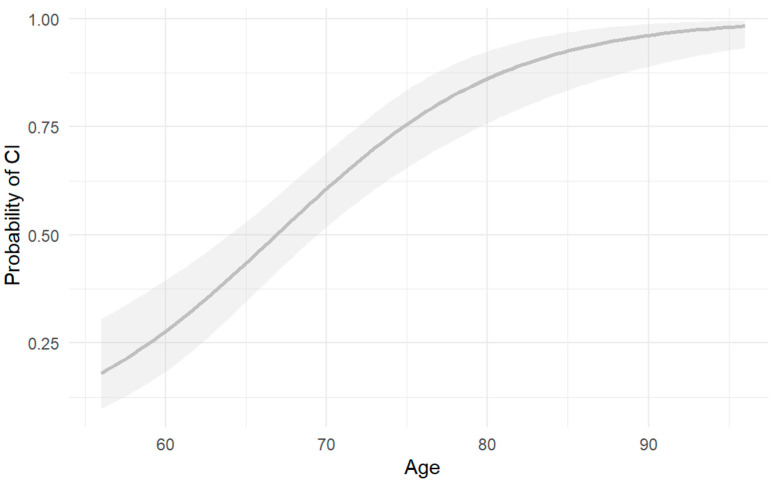
Risk of cognitive impairment according to age. Risk of CI as a function of number of years of age. The shaded area indicates CI for the depicted function OR = 1.149, CI = 1.094–1.21, *p* = 3.43 × 10^−8^, *p* < 0.001.

As previously mentioned, there were significant differences in age between the study groups, based on which logistic regression was performed. The relationship between age and cognitive impairment was evaluated using a logistic regression model, where statistically significant evidence was found, indicating that the probability of presenting with cognitive impairment as age increased was approximately 14.9% higher (OR = 1.149, CI = 1.094–1.21, *p* = 3.43 × 10^−8^) (Figure 3).

These results are consistent with those reported in the literature, in which the probability of cognitive impairment increases with age. As reported by a meta-analysis in Europe, the prevalence of Alzheimer’s disease increased with age, with a prevalence of 0.97, 7.66, and 22.53% in individuals aged 65–75 years, 75–84 years, and >85 years, respectively. Some studies conducted in Latino populations show the same pattern; the prevalence increases exponentially with age, doubling approximately every five years after age 65 [57].

Regarding genotype, 1.2% (*n* = 2) presented *APOE* ε2/ε3, 76.5% (*n* = 130) *APOE* ε3/ε3, 18.8% (*n* = 32) *APOE* ε3/ε4, 2.9% (*n* = 5) *APOE* ε4/ε4, and only 0.6% (*n* = 1) *APOE* ε2/ε4. For this analysis, the frequencies of individuals with cognitive impairment (CI) who were carriers and non-carriers of at least one ε4 allele were compared with those in the NCI-SMC group. Chi-square analysis revealed a significant association (χ^2^ = 4.009, *p* = 0.045). However, as shown in Table 2, the frequency of ε4 allele carriers was higher in the NCI-SMC group, whereas that of non-carriers was higher in the CI group. The mean age of carriers was slightly lower than that of non-carriers, and the years of education were slightly higher in the carrier group; however, these differences were not statistically significant (*p* = 0.966 and 0.347, respectively), suggesting a potential modulatory effect.

These results do not demonstrate a pattern consistent with the literature; therefore, it is possible that the influence of genotype was modified by clinical characteristics or lifestyle habits not considered in this analysis. It is important to consider that individuals with CI do not have a diagnosis of pure AD. Therefore, it is possible that the cognitive status of some individuals may have been influenced by mixed neurodegenerative pathologies.

Although it is widely recognized that the genotype is associated with an increased risk of developing the disease, various analyses have suggested that its effect does not act in isolation. Some publications have shown that the risk conferred by the *APOE* ε4 genotype varies by gender and age; a study by Molero et al. (2001) found that the presence of at least one ε4 allele was a significant age-stratified risk factor for Alzheimer’s disease only in women [58]. Other studies have found that the risk conferred by *APOE* ε4 for Alzheimer’s disease is higher in older men than in women, suggesting that both age and sex modulate the *APOE* ε4–Alzheimer’s disease association heterogeneously. Numerous studies have indicated that vascular pathology is an important risk factor for the development of cognitive impairment. A study by Oviedo et al. found that carotid stenosis was associated with worse cognitive performance in domains such as language, memory, attention, and visuospatial skills, independent of the *APOE* ε4 genotype [59].

Diabetes is another common metabolic disease that is associated with the development of neurodegenerative diseases. The presence of type 2 diabetes mellitus can increase the risk of dementia up to eight times, combined with stroke or independence, in addition to having the capacity to be the main cause of dementia in up to 43% of cases, as reported by Oviedo et al. [59].

This suggests that the development and progression of neurodegenerative pathologies are influenced, in addition to genotype, by other pathological processes that negatively affect brain function, and although there is no exclusive mechanism, cellular factors such as inflammatory responses, oxidative stress, low oxygen perfusion, and cerebrovascular disease have been proposed [60,61].

Several studies have shown that the *APOE* ε3 allele is the most common, whereas the *ε4* allele is associated with an increased risk of Alzheimer’s disease [62]. A meta-analysis of clinical studies reported that individuals carrying one copy of *ε4* had a 2.6–3.2 times higher risk of developing AD, whereas those with two copies had a 12.9 times higher risk [50,61].

Beyond metabolic factors, the study population should also be considered, because the influence and frequency of the *APOE* ε4 genotype seem to vary according to ethnicity. The literature mentions that *APOE* ε4 is less frequent in Mexican–American populations. For example, Campos et al. (2013) found that *APOE* ε4 frequencies were lower in Hispanic–Mexican controls than in non-Hispanic whites [63].

Not only does the frequency differ between different ethnic groups, but the risk has also been found to be variable, such as in Caucasian populations, where the *APOE* ε3/ε4 genotype appears to have a protective effect against Parkinson’s disease (PD) [64], whereas, in Asian populations, the ε4 allele and the *APOE* ε2/ε4 genotype were associated with an increased risk of PD [64].

Studies in Hispanic and Latino populations have shown racial variations in the magnitude of the association between the e4 allele and Alzheimer’s disease, with evidence of a smaller effect in individuals of non-European ancestry. Hispanic individuals of European ancestry have a stronger association between the *APOE* ε4 genotype and Alzheimer’s disease or other dementias than those of African or American Indian ancestry, such that the risk of e4 for cognitive impairment is only transmitted among non-Hispanic individuals [65].

Another study performed an analysis of Hispanic Americans of predominantly Caribbean origin, evidencing a lower risk of AD in relation to *APOE* ε4 in Hispanics than in non-Hispanic whites [66]. Ethnic differences have also been identified. Some studies have explored the association of *APOE* alleles through stratified analyses in different groups of origin, such as Cuban, Mexican, Puerto Rican, South American, and Central American. The results showed that the e4 allele is related to the risk for cognitive impairment in Central Americans, South Americans, and more strongly in Cubans, but no association was found in the Mexican group [67].

Similarly, an investigation conducted in a Mexican clinical population found no significant association between the *APOE* ε4 genotype and the presence of AD despite the higher frequencies of ε4 relative to the ε3 allele [68]. A meta-analysis by Huggins et al. showed that the association between the e4 allele and the risk of developing neurodegenerative diseases was statistically significant in Caribbean Hispanics, Central Americans, and Cubans, whereas no association was found in Mexican individuals [69].

In the present analysis, ancestry was not studied, in contrast with the *APOE* genotype results. However, given the ancestral composition of the Mexican population, with a higher proportion of indigenous–American ancestry (−45–55%) and a lower proportion of Europeans, mainly Spanish (30–45%), it is understandable that the association between cognitive impairment and the *APOE* ε4 allele does not reflect patterns widely reported in predominantly European populations [70]. It is important to consider that the high prevalence of vascular and metabolic comorbidities, such as diabetes, has a high incidence in Mexico (approximately 18.4%), almost five points above the world average, so that the mechanisms that trigger cognitive impairment are not reduced to the genotype [71,72].

The data obtained do not exclude the fact that the *APOE* ε4 genotype plays a causal role or is involved in the pathophysiological pathways of cognitive impairment but only emphasize ethnic differences, lifestyle, and comorbidities in populations.

### 2.3. Telomere Length and Cognitive Impairment

**Figure 4:**
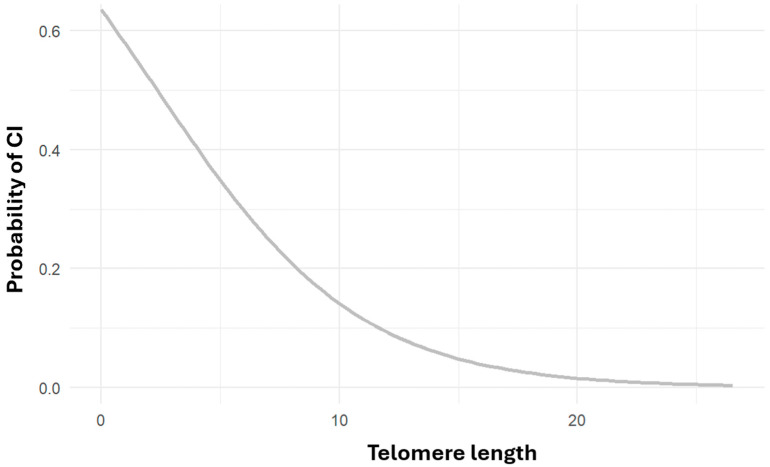
Risk of cognitive impairment as a function of telomeric length. Risk of cognitive impairment as a function of telomeric length number (*p* ≤ 0.05, OR = 0.789, CI = 0.6–1.01).

Logistic regression analysis revealed a statistically significant association between shorter telomere length and an increased risk of cognitive impairment (OR = 0.789, CI = 0.6–1.01, *p* < 0.05), suggesting that individuals with longer telomeres are approximately 21% less likely to exhibit cognitive impairment than those with shorter telomeres (Figure 4). Numerous studies have linked telomere shortening to elevated risks of cardiovascular disease, Alzheimer’s disease, and all-cause mortality. A recent systematic review concluded that individuals at higher risk for Alzheimer’s tend to have shorter telomeres, with Mendelian randomization studies supporting a potential causal relationship [73].

Crocco et al. (2023) found that individuals with Alzheimer’s disease had significantly shorter telomeres, independent of other risk factors, such as age, sex, and *APOE* ε4 genotype [74]. Other studies suggest a U-shaped relationship, in which both very short and very long telomeres are associated with an increased risk of Alzheimer’s disease [75].

Other authors have found a significant relationship between telomere length and the risk of Alzheimer’s disease, but with the behavior of U, inferring shorter and longer telomere lengths is associated with an increased risk of Alzheimer’s disease in the general population [76].

Telomeres are structures sensitive to the cellular and tissue microenvironment; therefore, their length is influenced by other variables such as physical exercise, smoking, alcoholism, and even the presence of comorbidities [77]. The mechanisms inherent to each variable are neither exclusive nor independently determined. However, it is known that oxidative stress is one of the main pathways related to length [78].

No statistically significant differences were found when evaluating the relationship between relative telomere length and the rest of the independent variables (sex and years of study), according to the simple logistic regression model. The findings reported in the literature are contradictory; some studies reported no significant differences between women and men, while others found that longer lengths were associated with better cognitive function only in women [79].

Smoking has also been linked to telomere length shortening; for example, a systematic review and meta-analysis of 83 studies concluded that current smokers have shorter telomeres than non-smokers and that there is an inverse relationship between the number of cigarettes consumed (measured in pack-years) and telomere length [80].

These findings support the hypothesis that smoking contributes to accelerated cellular aging and underscore the importance of interventions to reduce smoking as a preventive measure against age-related diseases. The scope of this study did not consider confounding variables such as physical exercise or smoking. However, the association observed between the patient groups with cognitive impairment and NCI-SMC suggests that, regardless of the cause of shortening, there is a relationship with an increased likelihood of cognitive impairment, and both smoking and sedentary lifestyles are known to contribute to neurodegenerative pathology [81].

### 2.4. Telomere Length and APOE Genotype

**Figure 5:**
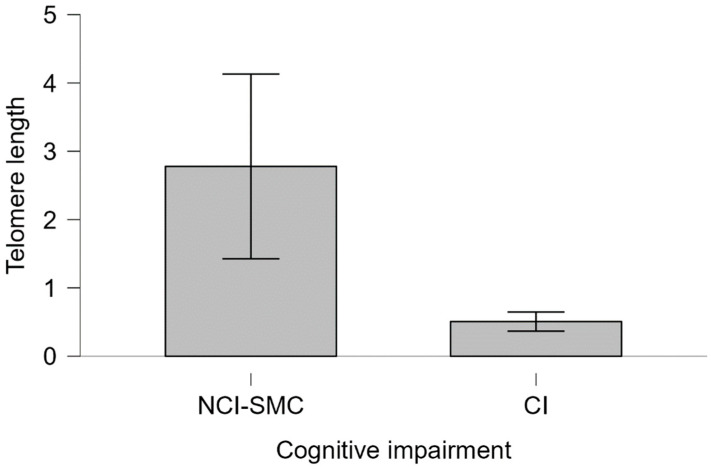
Telomeric length of *APOE* ε4 carriers and cognitive impairment Telomere length. Telomere length of individuals with CI and NCI-SMC carrying the ε4 allele is shown. The bars represent the standard error of the median for each group. Mann–Whitney 220.00, *p* = 0.116, Cohen’s d = 0.232, difference in means = −0.543, 95% CI (−1.561, 0.475).

**Figure 6:**
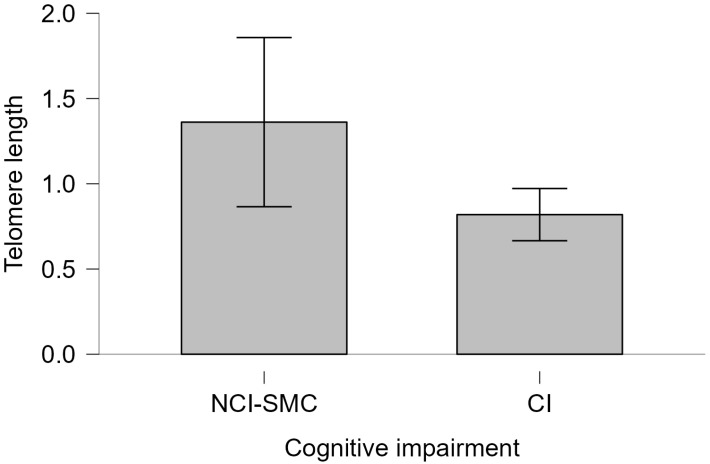
Telomeric length of *APOE* ε4 non-carriers and cognitive impairment of telomere length. Telomere length of individuals with CI and NCI-SMC not carrying the ε4 allele is plotted. The bars represent the standard error of the median for each group. Mann–Whitney 2075.5, *p* = 0.453, Cohen’s d = 0.012, difference in means = 0.4, 95% CI (–0.5 to 1.3).

No significant association was found between *APOE* ε4 status and telomere length (Kruskal–Wallis χ^2^ = 3.2385, df = 3, *p* = 0.3563). However, among *APOE* ε4 carriers, those with cognitive impairment showed a lower average telomere length than non-impaired carriers (Mann–Whitney U = 220.00, *p* = 0.116, Cohen’s d = 0.232) (Figure 5). In contrast, among non-carriers, no difference in telomere length was observed between individuals with and without cognitive impairment (Mann–Whitney U = 2075.5, *p* = 0.453, Cohen’s d = 0.012) (Figure 6).

Some researchers have found that individuals with genotypes other than *APOE* ε4 have longer telomeres, greater cerebral cortex thickness, and lower tau protein levels in CSF. Individuals with the *APOE* ε4 risk allele had a higher incidence of Alzheimer’s disease compared to individuals with a different genotype [77]. A study by Fani et al. found that the association between shorter telomeres and Alzheimer’s disease was stronger in individuals carrying *APOE* ε4, suggesting that cellular senescence caused by the short telomeric length of microglia may be a triggering mechanism for Alzheimer’s disease, which is exacerbated by amyloid protein aggregation [75].

The mechanisms underlying this relationship are not clear, but it is likely that oxidative stress and inflammatory mechanisms that have been described in Alzheimer’s disease due to the presence of amyloid aggregates are the pathways that link them. In this regard, the *APOE* ε4 genotype has shown increased platelet aggregation in the brains of cognitively impaired carriers, and according to several studies, oxidative stress is higher in this genotype than in individuals not carrying the allele. Synaptic protein levels are also altered in patients with Alzheimer’s disease, which triggers neuronal instability, metabolic stress, and increased neurodegeneration [82].

In addition, the dysfunction in glucose metabolism observed in patients with cognitive impairment and Alzheimer’s disease leads to a decrease in ATP levels. Consequently, loss of neuronal potential triggers excitotoxicity due to calcium accumulation. Eventually, the affected neurons die, and the brain volume decreases, causing cognitive alterations [82].

The rapid progression of neurodegeneration influenced by the *APOE* genotype may result in increased oxidative stress and cellular degeneration, thereby affecting telomere length maintenance. Although these mechanisms have not yet been fully established, elucidating these relationships enhances our understanding of the progression of neurodegeneration in neurocognitive disorders [83].

## 3. Materials and Methods

We included 170 individuals over 55 years from four healthcare centers. All participants were assessed using the Montreal Cognitive Assessment (MoCA) and Mini-Mental State Examination (MMSE) tests to determine their cognitive status, from which the following two study groups were formulated:

- Cognitive impairment group (CI): Individuals with cognitive impairment. All individuals with final scores below 25 points on the MoCA test and/or below 26 points on the MMSE test were included. Individuals with cognitive impairment due to non-neurodegenerative causes as established by the DSM-V (infections, delirium, and vitamin deficiency) were excluded. This study did not exclude participants with vascular or mixed neurocognitive disorder.
- Non-cognitive impairment with subjective memory complaint group (NCI-SMC): Individuals with subjective memory complaints but no cognitive impairment: Individuals who scored above the designated threshold for cognitive impairment on cognitive tests. It is important to consider that scores above 26 on the cognitive tests do not exclude the possibility of an initial decline in some cognitive domain reported by the participants; however, clinicians identified these cases as non-cognitive impairment. However, clinicians identified these cases as non-cognitive impairment.

The Mini-Mental State Examination and Montreal Cognitive Assessment tests were used for cognitive assessment. The MMSE excludes emotional and behavioral disorders and evaluates in 11 questions the temporospatial orientation, delayed memory, attention and calculation, language, and visuoconstructive drawing ability. The maximum possible score is 30 points, which is an indicator of normal cognition [84].

On the other hand, the MoCA test evaluates 6 cognitive domains: memory (5 points), visuospatial ability (4 points), executive function (4 points), attention/concentration/working memory (5 points), language (5 points), and orientation (6 points). The maximum sum of this test is 30 points, with a cut-off point for mild cognitive impairment of less than 26 points. In this test, the final score was adjusted for years of study, adding 1 point to individuals with less than 12 years of study and 2 points to those with less than 8 years of study [85].

All participants provided informed consent to participate in the study, and the collected clinical data and biological samples were coded to exclude any personal information, thereby safeguarding their privacy and identity.

Blood samples were collected for DNA extraction using the Gentra Puregene® kit (Qiagen, Hilden, Germany). The quality of the samples was analyzed by spectrophotometric quantification using a NanoDrop 2000 spectrophotometer (Thermo Fisher Scientific, Waltham, MA, USA).

Genotyping of *APOE* gene polymorphisms (rs7412 and rs429358) was performed by real-time PCR (rt-PCR) using TaqMan^®^ probes (Applied Biosystems, San Francisco, CA, USA). For the genotyping of the rs429358 and rs7412 polymorphisms, assays C_3084793_20 and C_904973_10 were used, respectively. The thermocycling conditions followed the manufacturer’s recommendations for each assay. Thermocycling and allelic discrimination were performed using the QuantStudio 6 Flex^®^ real-time instrument (Applied Biosystems, San Francisco, CA, USA).

To determine telomere length, rt-PCR was conducted using a QuantStudio 6 Flex system (Thermo Fisher Scientific, Waltham, MA, USA). Standard curves were generated, and TL data were expressed using the 2^−∆∆CT^ method, based on telomere threshold cycle (TC) values and reference gene signals (∆CT). The control gene used was SDHA (succinate dehydrogenase complex flavoprotein subunit A).

### Statistical Analysis

Statistical comparisons were performed only between the two defined clinical groups (CI and NCI-SMC). The chi-square test was used to evaluate the association of the *APOE* genotype between both groups, comparing *APOE* ε4 allele frequencies with the remaining *APOE* ε2 and *APOE* ε3. Multivariate linear and logistic regression models were used to assess the relationships between telomere length, age, and years of education with cognitive impairment. All analyses were performed using RStudio software (version 2024.04.1).

## 4. Conclusions

Shorter telomere length was associated with an increased risk of cognitive decline. Similarly, as the strongest predictor in this study, fewer years of education were associated with an increased likelihood of cognitive decline, supporting the role of cognitive reserve as a possible protective factor, independent of genotype, age, and sex. Furthermore, although the *APOE* ε4 genotype was not directly associated with an increased risk of cognitive decline, it may contribute to telomere shortening.

## Figures and Tables

**Table 1:** Sociodemographic and clinical characteristics of subjects with CI and NCI-SMC.

	Mean (*n* = 170, %)	CI (99, 58.24)	NCI-SMC (71, 41.76)
**Age ^a^**	70.5 (±9.224)	74.18 (±9.12)	65.36 (±6.57)
**Years of study ^a^**	12.55 (±6.318)	9.45 (±5.77)	16.88 (±4.13)
**Female ^b^**	94 (55.29)	47 (27.65)	47 (27.65)
**Male ^b^**	76 (44.7)	52 (30.58)	24 (14.11)
**MMSE ^a^**	21.31 (±7.03)	16.85 (±5.72)	28 (±1.33)
**MoCA ^a^**	24.63 (±3.87)	21.56 (±3.6)	27.23 (±1.41)
**Non-carriers *APOE* ε4 ^b^**	131 (77.65)	82 (48.23)	49 (28.82)
**Carriers *APOE* ε4 ^b^**	39 (22.35)	17 (10)	22 (12.94)
**Telomere length (2** **^−^** **^ΔCT^** **) ^a^**	1.04 (±2.38)	0.76 (±1.28)	1.43 (±3.36)

CI: cognitive impairment; NCI-SMC: non-cognitive impairment with subjective memory complaints. ^a^ Mean and standard deviations are shown in parentheses. ^b^ The frequency and percentage in parentheses calculated from the total sample.

**Table 2:** Genotype and cognitive impairment.

	*APOE* ε4		
	With	Without	*p*-Value
**Cognitive impairment ^a^**	**17 (10)**	**82 (48.23)**	*p* < 0.05 *
Age ^b^	74.118 (±8.49)	74.195 (±9.29)	
Years of study ^b^	7.941 (±5.55)	9.964 (±6.01)	
**NCI-subjetive memory complaint ^a^**	**22 (12.94)**	**49 (28.8)**	
Age ^b^	63.238 (±5.04)	66.26 (±6.56)	
Years of study ^b^	18.047 (±5.8)	18.204 (±5.53)	
**Total ^a^**	**39 (22.94)**	**132 (77.65)**	
Age	68.105 (±8.67)	71.189 (±9.3)	*p* = 0.966
Years of study	13.52 (±7.58)	13.023 (±7.05)	*p* = 0.347

^a^ Frequency is shown, and in parentheses, the percentage of individuals calculated with the total n. ^b^ The mean is shown, and in parentheses, the standard deviation for each group. * The *p*-values were calculated by comparing the variables of cognitive impairment, age, and years of study between individuals carrying and not carrying the e4 allele.

## Data Availability

Data are contained within the article.
